# Variation in Mpox Vaccine Coverage in the United States: Influence of Political Orientation and Public Health Resources

**DOI:** 10.1093/ofid/ofae567

**Published:** 2024-09-26

**Authors:** HeeEun Kang, Suzette Rovelsky, Nam Hoon Kang, Elizabeth A Talbot, Richard A Murphy

**Affiliations:** Section of Infectious Disease and International Health, Department of Medicine, Dartmouth Hitchcock Medical Center, Lebanon, New Hampshire, USA; Department of Medicine, Geisel School of Medicine at Dartmouth, Hanover, New Hampshire, USA; Department of Medicine, Geisel School of Medicine at Dartmouth, Hanover, New Hampshire, USA; Department of Economics, Hanshin University, Osan, Republic of Korea; Section of Infectious Disease and International Health, Department of Medicine, Dartmouth Hitchcock Medical Center, Lebanon, New Hampshire, USA; Department of Medicine, Geisel School of Medicine at Dartmouth, Hanover, New Hampshire, USA; Department of Medicine, Geisel School of Medicine at Dartmouth, Hanover, New Hampshire, USA; Infectious Disease Section, Department of Medicine, White River Junction Veterans Affairs Medical Center, White River Junction, Vermont, USA

**Keywords:** emerging infections, emergency preparedness, sexually transmitted infection, vaccine campaign

## Abstract

During the 2022–2023 mpox outbreak in the United States, the federal government distributed mpox vaccines to individual states. Significant variation in vaccine coverage was noted across states. State-level factors associated with higher vaccine coverage were Democratic political orientation, higher public health spending, higher proportion of at-risk population, and higher mpox cases.

Mpox (previously monkeypox) is an orthopoxvirus endemic in Central and West Africa that causes a spectrum of human disease ranging from rash and febrile illnesses to death [1]. The 2022–2023 mpox epidemic in the United States (US) started with the first case identified in May 2022 in Massachusetts. Since then, >32 000 infections have been identified nationally, and ongoing low levels of transmission continue [2]. Unlike the coronavirus disease 2019 (COVID-19) pandemic, there was already a US Food and Drug Administration–approved mpox vaccine (JYNNEOS) available at the onset of this mpox outbreak. This vaccine was stockpiled in large quantities in the Strategic National Stockpile as part of the US emergency preparedness effort [3]. As a response to the outbreak, the US federal government distributed the vaccines from the stockpile to states and jurisdictions to provide to at-risk persons. As of 9 January 2024, close to 1.3 million doses of mpox vaccine were administered in US jurisdictions; the majority of the vaccinations occurred between July and December 2022 [4]. There was considerable geographical variation in early access to vaccines, with different states achieving different rates of vaccine coverage [5]. This variability persisted into 2024 [4].

The mpox vaccine was not initially commercially available, and state public health systems were fully responsible for distribution of these vaccines to at-risk individuals. In the US, the public health infrastructure and the healthcare delivery system intersect in many ways but are separate: the former being the purview of jurisdictional governments with a focus on public health and safety, and the latter being largely based in the private sector with a focus on delivering medical care for individuals [6]. States have different public health structures with varying amounts of funding, staffing, and physical infrastructure. For example, New Hampshire is a rural state with a centralized public health structure with only 2 freestanding city public health departments (Manchester and Nashua). To deliver mpox vaccines in New Hampshire to individuals living outside of these 2 city areas, the New Hampshire Department of Health and Human Services had to create partnerships with private medical facilities to establish public vaccine clinics [7]. Patients using these private facilities in New Hampshire may have had to pay for mpox vaccine administration, though the vaccine itself came at no cost to patients.

Given the exclusive distribution role local public health played during the mpox vaccine campaign, local public health capacity and funding likely influenced regional vaccine coverage. In the literature, variable vaccine coverage was thought to be due to differences in vaccine awareness, lack of vaccine providers, lack of vaccine confidence and demand, and stigma [5]. Inequities based on race and ethnicity have also been documented: Black and non-Hispanic American Indian or Alaska Native persons are less likely to have received the mpox vaccine [8, 9], which may be related to the geographical variation.

We conducted an analysis using available public data to identify state-level factors associated with variation in mpox vaccine coverage. We hypothesized that the state's political party orientation, public health funding, public health staffing, diversity index (probability that 2 persons chosen at random will be from different racial and ethnic groups), poverty rate, proportion of at-risk population, and mpox cases would be significantly associated with mpox vaccine coverage.

## METHODS

We collected publicly available data on mpox vaccine coverage by state and jurisdiction from the Centers for Disease Control and Prevention (CDC) (data updated 28 November 2023) [4]. New York City and Philadelphia were combined with New York state and Pennsylvania, respectively. Washington, DC, was treated as a state for the analysis, and Puerto Rico was not included. Complete vaccination was defined as an individual having received 2 doses of the mpox vaccine. Political party orientation of each state was determined by the 2020 presidential election result (electoral) [10]. Each state's public health funding per capita and staffing was obtained from the Association of State and Territorial Health Officials (ASTHO) Profile of the State online publication, using data from 2021. Public health staffing was total full-time equivalent (filled and unfilled) per 100 000 population per ASTHO [11]. US Census data were used for the diversity index [12] and the poverty rate (data from 2020) [13].

The proportion of persons considered at-risk within the state population was calculated by dividing the number of estimated at-risk persons per state by the total population of that jurisdiction based on the ASTHO publication. The estimated at-risk persons per state was obtained from the CDC mpox vaccine coverage dataset, noted as MSM++: the number of men who have sex with men (MSM) with an indication to receive human immunodeficiency virus (HIV) preexposure prophylaxis (≥16 years of age), plus the number of MSM with HIV (≥13 years of age) using data from CDC AtlasPlus [5]. Mpox case counts were obtained from the CDC website (data updated 28 November 2023) [4].

Univariable and multivariable linear regression analyses were performed using R software (version 4.3.2) [14]. *P* values of <.05 were used to establish a significant correlation. The appropriateness of the linear regression model was checked using the Ramsey regression equation specification error (RESET) test.

## RESULTS

Across all states, mpox vaccine coverage for at-risk persons was 21%. Nationally, public health spending per person averaged US$162, and public health staffing averaged 43 full-time equivalent per 1000 persons. Average diversity index was 49%, and the poverty rate was 11%. Proportion of MSM++ per 1000 people averaged 5.2, and mpox cases by state averaged 609 (Table 1). The full dataset is available under Supplementary Table 1.

**Table 1.** Mpox Vaccine Coverage and Associated Factors for US States

Factor	Average (Mean)	Lowest	Highest
Vaccination coverage	21%	WV (5%)	DC (68%)
Politics: Republican (n = 25)	14%	WV (5%)	UT (35%)
Politics: Democrat (n = 26)	28%	MD, NH (12%)	DC (68%)
Public health spending ($US per capita)	$162	TX ($27)	HI ($691)
Public health staffing (total FTE per 1000)	43	NY, MI (7)	NM (189)
Diversity index	49%	ME (19%)	HI (76%)
Poverty rate	11%	NH (5%)	MS (19%)
At-risk population (MSM++ per 1000)	5.2	SD (1.5)	DC (32.4)
Mpox cases, No.	609	SD, VT (3)	CA (6015)

Abbreviations: FTE, full-time equivalent; MSM++, men who have sex with men (MSM) with an indication to receive human immunodeficiency virus (HIV) preexposure prophylaxis (≥16 years of age), plus the number of MSM with HIV (≥13 years of age); US, United States.

State abbreviations: CA, California; DC, Washington, District of Columbia; NH, New Hampshire; HI, Hawaii; MD, Maryland; ME, Maine; MI, Michigan; MS, Mississippi; NM, New Mexico; NY, New York; SD, South Dakota; TX, Texas; UT, Utah; VT, Vermont; WV, West Virginia.

Univariable linear regression analysis showed that Republican political orientation was associated with lower mpox vaccine coverage (−13.4% lower vaccine coverage compared to Democratic states; *P* < .001). Factors associated with higher vaccine coverage included the following: increased public health spending (US$1 increased spending associated with 0.048% higher vaccine coverage; *P* = .002), increased diversity index (1% increase in diversity index associated with 0.240% higher vaccine coverage, *P* = .039), increased proportion of at-risk population (each additional MSM++ per 1000 associated with 1.715% higher vaccine coverage, *P* < .001), and higher mpox cases (each additional mpox case associated with 0.003% higher mpox vaccine coverage, *P* = .028) (Table 2).

**Table 2.** Factors Associated With Mpox Vaccine Coverage: Linear Regression

Factor	Univariable		Multivariable	
	Coefficient (95% CI)	*P* Value	Coefficient (95% CI)	*P* Value
Politics: Republican	−13.39 (−19.08 to −7.70)	**<**.**001**	−5.65 (−10.90 to −.40)	**<**.**036**
Public health spending ($ per capita)	.048 (.014–.076)	.**002**	.053 (.027–.079)	**<**.**001**
Public health staffing (total FTE per 1000)	.067 (−.013 to .148)	.100	−.073 (−.148 to .002)	.055
Diversity index (%)	.240 (.013–.468)	.**039**	−.091 (−.280 to .098)	.337
Poverty rate (%)	−.976 (−2.180 to .228)	.110	−.906 (−1.826 to .014)	.053
At-risk population (MSM++ per 1000)	1.715 (1.066–2.364)	**<**.**001**	1.529 (.939–2.120)	**<**.**001**
Mpox cases	.003 (.000–.006)	.**028**	.003 (.000–.005)	.**032**

Values in bold indicate statistical significance.

Abbreviations: CI, confidence interval; FTE, full-time equivalent; MSM++, men who have sex with men (MSM) with an indication to receive human immunodeficiency virus (HIV) preexposure prophylaxis (≥16 years of age), plus the number of MSM with HIV (≥13 years of age).

In multivariable linear regression analysis using all studied variables, the following factors remained significantly associated with vaccine coverage: Republican states achieved 5.65% lower vaccine coverage (*P* = .036), each US$1 increase in public health spending increased vaccine coverage by 0.05% (*P* < .001), each additional MSM++ per 1000 people increased vaccine coverage by 1.5% (*P* < .001), and each additional mpox case increased vaccine coverage by 0.003% (*P* = .032) (Table 2). Multicollinearity check showed low correlation among tested variables with variance inflation factor ranging from 1.18 to 1.64. Ramsey RESET test resulted in *P* = .161, indicating the appropriateness of a general linear regression model.

## DISCUSSION

There was significant variation in full mpox vaccine coverage across states during the US mpox outbreak of 2022–2023. The strongest factor associated with full mpox vaccine coverage identified in our study was the political orientation of the state, with Republican states achieving significantly lower rates of vaccination even after adjusting for public health funding, public health staffing, diversity index, poverty rate, proportion of MSM++ in the population, and mpox case counts. What could be the mechanism behind this correlation between political orientation and mpox vaccine uptake?

Stigma is a potential social mechanism contributing to the relationship between state politics and mpox vaccine uptake. Stigma was the most commonly identified theme regarding mpox—mentioned by 91.6% of interviewees—in an interview study with 24 Black sexual minority men living in the Washington, DC metropolitan area [15]. Stigma was noted to be an important driver of mpox vaccine hesitancy in qualitative studies [15, 16]. Republican states’ political climate may have contributed to stigma against sexual minority men, leading to lower vaccine uptake. Another potential mechanism is variation in the level of provider trust. Trust in one's doctor and healthcare provider is associated with lower mpox vaccine hesitancy [17]. Adolescent sexual minority men living in Republican states are less likely to have a discussion with medical provider about sex with males, condom use with male partners, and HIV testing [18], perhaps in part due to a lower level of trust and stigma. HIV outcomes including age-standardized mortality rate and case-fatality rates are highest in the South [19], where most states are Republican.

Vaccine hesitancy was a major barrier for the COVID-19 vaccine campaign and likely contributed to lower mpox vaccine uptake rates. Prominent reasons for COVID-19 vaccine hesitancy included being generally anti-vaccination, concerns about safety and rushed vaccine development, doubts about need for the vaccine, and doubts about vaccine efficacy, among others [20]. Notably, Republicans reported higher COVID-19 vaccine hesitancy compared to Democrats [21]. Yet the vast majority of sexual minority men identify as Democrats [22], and sexual minority men are more likely than the general population to have received the COVID-19 vaccine [23]. For the mpox vaccine, safety had been already established and the US Food and Drug Administration approval process was not (and did not appear to be) rushed. Concern for stigma, lack of knowledge of disease, and lack of self-perceived risk were identified as drivers of mpox vaccine hesitancy [15, 16] rather than concerns regarding vaccine safety, rapid vaccine development, or efficacy. We were unable to find definitive proof that politics drove mpox vaccine hesitancy. This is an area that warrants further research.

Another possible reason for the variation we noted may be differential vaccine availability in Republican versus Democratic states. Local public health entities were responsible for distributing and delivering vaccines to at-risk persons. Individual health departments have differing funding allocated to sexually transmitted infections or emergency preparedness, and different competing public health priorities. The existing public health infrastructure (such as easily accessible public health clinics), the size of at-risk population living in the state, and local political climate may have affected how intensely each state's public health organizations focused on the mpox vaccine distribution efforts. Inequitable vaccine availability is an important problem that goes beyond the US. There was considerable inequity in the global distribution of mpox vaccines worldwide, with endemic countries in West and Central Africa experiencing severe supply shortages [24]. Further studies and advocacy work are needed to help alleviate such disparate access.

Our study highlights the importance of sustained investment into public health by increasing funding, staffing, and infrastructure. States that had higher funding per capita performed better even when controlling for the diversity index and the poverty rate. The funding estimates from the mpox public vaccine clinic establishment in the New Hampshire survey study reported modest needs (average $2057 per month estimated by responding facilities) [7]. Our study highlights the need to develop strategies to rapidly support each state's public health infrastructure for public vaccine distribution, especially in an emergency outbreak setting when no concurrent private-sector measures can be taken.

The strengths of our study include its reliance on public data, reproducibility, and our inclusion of multiple factors that may influence vaccine uptake. This study has several limitations. We focused on mpox vaccine coverage as the outcome and not the number of mpox cases, which some may consider to be the more meaningful outcome. However, it is important to highlight gaps in preventive measures and to look for ways to improve public vaccination responses. Our analysis is limited by the accuracy of available public data: for instance, there may be inaccuracies in the CDC estimate of proportion of at-risk population (MSM++) per state. We categorized each state's political identity as a binary variable, yet recognize that state-level political orientation is more complex than the result from one presidential election. We did not include all federal agencies such as the Department of Veterans Affairs and the Indian Health Service, which received separate supplies of mpox vaccines by the federal government. Given the observational nature of the study, we are unable to conclusively comment on causality of the relationships identified nor definitively identify the mechanism through which the correlations are seen. Regardless, our work highlights an important area for improvement: delivering equitable preventive care during a smaller outbreak affecting a stigmatized population. Our work is particularly relevant for public health, nongovernmental, and community organizations in Republican states to be aware of this potential problem, and act early and assertively to advocate for vaccination opportunities for sexual minority men.

## CONCLUSIONS

Higher mpox vaccine coverage was achieved in US states with Democratic political orientation, higher public health funding, higher proportion of at-risk population, and higher number of mpox cases during the mpox outbreak of 2022–2023. Equitable access to disease-preventing vaccines, particularly in an outbreak setting, is of critical importance.

## Supplementary Material

ofae567_Supplementary_Data

## Supplementary Data

Supplementary materials are available at *Open Forum Infectious Diseases* online. Consisting of data provided by the authors to benefit the reader, the posted materials are not copyedited and are the sole responsibility of the authors, so questions or comments should be addressed to the corresponding author.

## Notes

***Author contributions.*** H. K. conceptualized and designed the study, collected data, performed data analysis, and drafted and revised the manuscript. S. R. and E. A. T. helped design the study and reviewed and revised the manuscript. N. H. K. performed data analysis and provided statistical support. R. A. M. reviewed the data analysis and reviewed and revised the manuscript.
