# Supplementary material for: Variation in Mpox Vaccine Coverage in the United States: Influence of Political Orientation and Public Health Resources
Source: Open Forum Infect Dis. 2024 Sep 26;11(10):ofae567. doi: 10.1093/ofid/ofae567 (PMC11475597; doi:10.1093/ofid/ofae567)
Supplement: ofae567_Supplementary_Data [file ofae567_supplementary_data.docx]

Supplementary Index

Table 1. Raw data table

| **State** | **Politics** | **Vaccine coverage (%)** | **Public health spending ($)** | **Public health staffing** | **Diversity index (%)** | **Poverty rate (%)** | **At-risk per 1,000** | **Mpox cases** |
| --- | --- | --- | --- | --- | --- | --- | --- | --- |
| Alabama | R | 7 | 125 | 49 | 53.1 | 14.6 | 4.25 | 193 |
| Alaska | R | 22 | 311 | 46 | 62.8 | 12.2 | 3.13 | 5 |
| Arizona | D | 20 | 146 | 21 | 61.5 | 11.2 | 5.80 | 597 |
| Arkansas | R | 7 | 139 | 67 | 49.8 | 14.7 | 3.32 | 79 |
| California | D | 42 | 130 | 12 | 69.7 | 11.0 | 7.44 | 6,015 |
| Colorado | D | 28 | 238 | 31 | 52.3 | 9.3 | 6.44 | 418 |
| Connecticut | D | 32 | 172 | 23 | 55.7 | 9.9 | 3.97 | 148 |
| Delaware | D | 21 | 209 | 92 | 59.6 | 8.1 | 6.35 | 44 |
| District of Columbia | D | 68 | 425 | 173 | 67.2 | 14.6 | 32.38 | 549 |
| Florida | R | 20 | 120 | 46 | 64.1 | 12.8 | 9.28 | 2,923 |
| Georgia | D | 23 | 103 | 12 | 64.1 | 13.4 | 7.83 | 2,034 |
| Hawaii | D | 29 | 691 | 167 | 76 | 9.5 | 4.54 | 39 |
| Idaho | R | 12 | 98 | 13 | 35.9 | 9.2 | 3.11 | 17 |
| Illinois | D | 32 | 75 | 13 | 60.3 | 9.2 | 6.59 | 1,557 |
| Indiana | R | 9 | 80 | 13 | 41.3 | 11.2 | 4.55 | 300 |
| Iowa | R | 21 | 80 | 13 | 30.8 | 9.1 | 2.18 | 30 |
| Kansas | R | 12 | 142 | 62 | 45.4 | 8.7 | 2.68 | 50 |
| Kentucky | R | 13 | 131 | 9 | 32.8 | 14.4 | 4.29 | 109 |
| Louisiana | R | 19 | 126 | 27 | 58.6 | 17.4 | 6.27 | 318 |
| Maine | D | 22 | 106 | 26 | 18.5 | 10.0 | 3.79 | 13 |
| Maryland | D | 12 | 74 | 124 | 67.3 | 8.1 | 7.21 | 757 |
| Massachusetts | D | 39 | 217 | 43 | 51.6 | 8.2 | 5.28 | 471 |
| Michigan | D | 14 | 118 | 7 | 45.2 | 10.6 | 4.28 | 399 |
| Minnesota | D | 25 | 206 | 36 | 40.5 | 7.3 | 4.93 | 246 |
| Mississippi | R | 6 | 151 | 63 | 55.9 | 18.8 | 3.81 | 109 |
| Missouri | R | 11 | 286 | 27 | 40.8 | 10.8 | 4.77 | 225 |
| Montana | R | 10 | 100 | 20 | 30.1 | 10.3 | 2.63 | 7 |
| Nebraska | R | 29 | 150 | 20 | 40.8 | 9.2 | 2.04 | 34 |
| Nevada | D | 22 | 91 | 14 | 68.8 | 12.1 | 6.91 | 334 |
| New Hampshire | D | 12 | 108 | 22 | 23.6 | 5.3 | 2.86 | 35 |
| New Jersey | D | 19 | 175 | 64 | 65.8 | 7.6 | 4.70 | 787 |
| New Mexico | D | 21 | 338 | 189 | 63 | 16.2 | 4.90 | 57 |
| New York | D | 42 | 112 | 7 | 65.8 | 11.8 | 7.51 | 4365 |
| North Carolina | R | 19 | 76 | 9 | 57.9 | 13.2 | 5.35 | 711 |
| North Dakota | R | 15 | 114 | 27 | 32.6 | 9.5 | 2.33 | 6 |
| Ohio | R | 15 | 92 | 10 | 40.4 | 12.3 | 5.00 | 403 |
| Oklahoma | R | 9 | 122 | 59 | 59.5 | 13.2 | 4.15 | 83 |
| Oregon | D | 29 | 156 | 27 | 46.1 | 9.1 | 6.20 | 299 |
| Pennsylvania | D | 20 | 45 | 11 | 44 | 10.4 | 4.35 | 872 |
| Rhode Island | D | 46 | 398 | 49 | 49.4 | 8.8 | 5.04 | 85 |
| South Carolina | R | 9 | 121 | 54 | 54.6 | 13.7 | 4.41 | 239 |
| South Dakota | R | 14 | 198 | 38 | 35.6 | 10.9 | 1.49 | 3 |
| Tennessee | R | 10 | 169 | 40 | 46.6 | 12.8 | 5.21 | 406 |
| Texas | R | 9 | 27 | 11 | 67 | 12.9 | 6.91 | 3,085 |
| Utah | R | 35 | 138 | 36 | 40.7 | 7.2 | 2.96 | 200 |
| Vermont | D | 31 | 260 | 100 | 20.2 | 9.0 | 2.55 | 3 |
| Virginia | D | 19 | 108 | 35 | 60.5 | 8.8 | 5.62 | 576 |
| Washington | D | 27 | 100 | 49 | 55.9 | 7.9 | 6.76 | 726 |
| West Virginia | R | 5 | 138 | 45 | 20.2 | 14.6 | 3.69 | 12 |
| Wisconsin | D | 21 | 102 | 8 | 37 | 8.3 | 3.10 | 93 |
| Wyoming | R | 16 | 130 | 47 | 32.4 | 9.5 | 2.02 | 4 |
| Abbreviations: D = Democrat, R = Republican | | | | | | | | |
